# Multi-Level Stigma, Discrimination, and Mental Health Care Barriers Among Brazilian Youth: A Scoping Review

**DOI:** 10.3390/healthcare14213616

**Published:** 2026-10-26

**Authors:** Bianca Teeny Sallum, Maria Gabriela Custodio de Figueiredo, Monaliza Ehlke Ozorio Haddad, Tiago S. Bara, Vanessa Furlin, Mara L. Cordeiro

**Affiliations:** 1 Escuela de Doctorado, Universidad de Salamanca, 37008 Salamanca, Spain; idu054400@usal.es; 2 Instituto de Pesquisa Pelé Pequeno Príncipe, Curitiba 80250-060, PR, Brazil; gabi.figueiredo@mail.utoronto.ca (M.G.C.d.F.); tiago.bara@pelepequenoprincipe.org.br (T.S.B.); vanessa.furlin@pelepequenoprincipe.org.br (V.F.); 3 Instituto Aprendizagem e Desenvolvimento (IAD), Araucária 83702-080, PR, Brazil; monalizahaddad@iadpr.com.br; 4 Faculdades Pequeno Príncipe, Curitiba 80230-020, PR, Brazil; 5 Department of Psychiatry and Biobehavioral Sciences, David Geffen School of Medicine, University of California, Los Angeles, Los Angeles 90025, CA, USA

**Keywords:** mental health stigma, adolescents, Brazil, health equity, help-seeking behavior, depressive disorders

## Abstract

**Highlights:**

**What are the main findings?**

• Stigma and discrimination were associated with adverse mental health outcomes among Brazilian youth, with the most consistent evidence relating to depressive symptoms and depression; most estimates derived from mixed adolescent and young-adult samples.
• Qualitative evidence identified stigma-related barriers to help-seeking, disclosure, diagnostic processes, and engagement with mental health care.

**What are the implications of the main findings?**

• Stigma-informed approaches may support earlier engagement with mental health care among youth exposed to racial, sexual orientation, or gender identity discrimination, although their effectiveness requires evaluation.
• Major evidence gaps remain, including limited longitudinal research, culturally validated stigma measures, and representation from Brazil’s North and Midwest regions.

**Abstract:**

**Background/Objectives:** To map mental illness-related stigma and identity-based stigma and discrimination in relation to depression, anxiety, and mental health care processes among Brazilian youth. **Methods:** This scoping review followed PRISMA-ScR guidelines and the Arksey and O’Malley framework. PubMed/MEDLINE, Scopus, SciELO, LILACS, and CAPES were searched for studies examining stigma in relation to depression, anxiety, mental health outcomes, or care-related processes among Brazilian children and adolescents. Mixed adolescent and young-adult samples were included when they contained adolescents within the target age range. **Results:** Seven studies met the inclusion criteria. Stigma was identified across four domains: public, structural, self-, and courtesy stigma. Perceived discrimination was associated with major depression (aPR = 2.00, 95% CI 1.37–2.97) and severe depressive symptoms (aOR = 1.71, 95% CI 1.23–2.38) in studies examining racial discrimination and discrimination affecting sexual and gender minority youth. These estimates were derived from samples that included young adults. Qualitative evidence linked stigma to symptom concealment, barriers to help-seeking and diagnostic processes, and difficulties engaging with care. Evidence was limited by predominantly cross-sectional and qualitative designs, uneven geographic representation across Brazil, and a lack of culturally validated stigma measures. **Conclusions:** Stigma appears to be a multi-level factor associated with adverse mental health outcomes and with reported barriers across the mental health care pathway among Brazilian youth. Stigma-informed approaches may support earlier engagement with care among marginalized youth, but their effectiveness remains to be evaluated.

## 1. Introduction

Adolescent depression and anxiety are among the leading causes of disability worldwide, with early onset frequently associated with persistent impairments in social, academic, and functional outcomes [1,2]. In low- and middle-income countries (LMICs), including Brazil, these conditions are further shaped by profound socioeconomic inequalities and uneven access to mental health services within public health systems [3,4]. While the burden of these disorders is well documented, less attention has been given to the mechanisms through which social disadvantage contributes to adverse mental health outcomes and disrupted care trajectories [5].

Stigma is conceptualized as a social process in which labeling, stereotyping, separation, status loss, and discrimination converge within unequal power relations [6]. In this review, stigma encompasses both mental illness-related stigma and discrimination based on marginalized social identities, including race, sexual orientation, and gender identity. Stigma has been increasingly recognized as a key factor in this process, operating across individual and institutional levels to influence mental health outcomes and engagement with care [6,7]. International evidence suggests that stigma is associated with greater symptom severity, reduced help-seeking, and poorer treatment engagement among adolescents [8,9]. Fear of social rejection, ridicule, and labeling may further discourage disclosure, promote symptom concealment, and reduce help-seeking among adolescents [9,10,11,12].

Beyond individual experiences, stigma also operates at a structural level, shaping access to care and exposure to stress [7,13]. In Latin American settings, these processes are further influenced by cultural dynamics such as familial secrecy and stigma by association, which shape how mental illness is interpreted, managed, and often concealed within family systems [5].

In Brazil, the literature has extensively documented the role of broader social determinants, including violence, racism, and socioeconomic inequality, in shaping youth mental health [14,15]. However, stigma has often been treated as an implicit or secondary construct rather than as a distinct pathway linking social inequities to adverse mental health outcomes. This gap is further reinforced by what we characterize as a measurement gap reflecting the limited availability of culturally validated instruments capable of capturing local linguistic and conceptual distinctions, such as the differentiation between “preconceito” (prejudice) and “estigma” (stigma) [3,16]. As a result, robust indicators for monitoring stigma and informing policy remain limited.

To address these gaps, this scoping review aims to map mental illness-related stigma and identity-based stigma and discrimination associated with depression and anxiety among children and adolescents in Brazil. Specifically, it maps the evidence on associations between stigma and clinical outcomes, including symptom severity and suicidality, as well as care-related processes such as help-seeking, diagnosis, access, and engagement with care. By synthesizing national evidence and situating it within the broader international literature, this review aims to identify context-specific challenges and shared patterns, contributing to a broader understanding of stigma and mental health inequities among Brazilian adolescents.

## 2. Materials and Methods

### 2.1. Study Design

This scoping review was conducted in accordance with the methodological framework proposed by Arksey and O’Malley [17], further refined by Levac et al. [18], and reported following the Preferred Reporting Items for Systematic Reviews and Meta-Analyses Extension for Scoping Reviews (PRISMA-ScR) guidelines [19]. The objective was to map mental illness-related and identity-based stigma and discrimination associated with depression and anxiety among Brazilian youth, examining mental health outcomes and care-related processes. A predefined review protocol specifying eligibility criteria, search strategies, and data extraction domains was deposited on the Open Science Framework (OSF; registration: zr29e) on 12 April 2026, before the initial database search exports. Formal registration was initiated on 13 April 2026, preceding the start of study screening on 14 April 2026. The registration was approved on 16 April 2026 under a time-limited embargo, which ended on 30 June 2026 (https://osf.io/zr29e accessed on 13 October 2026).

### 2.2. Eligibility Criteria and Types of Evidence Sources

Eligibility criteria were defined using the Population–Concept–Context (PCC) framework. The population included children and adolescents under 18 years of age residing in Brazil. Studies with mixed adolescent and young-adult samples were also eligible when they included adolescents within the target age range; adult-only populations were excluded. When age-stratified results were unavailable, findings from the full analytic sample were extracted as reported in the original study. The concept encompassed mental illness-related stigma and identity-based stigma or discrimination (e.g., racial, sexual orientation, and gender identity), examined in relation to depression, anxiety, associated outcomes such as suicidality, or care-related processes including help-seeking, access, diagnosis, treatment, and engagement. The context encompassed studies conducted in Brazil across community, educational, and healthcare settings, including both public and private health services and all geographic regions.

Eligible studies included quantitative observational designs (cross-sectional, cohort, and retrospective analyses), qualitative studies, mixed-methods studies, and intervention studies related to diagnostic pathways or provider training. We searched for grey literature (e.g., theses and dissertations) but found no relevant studies. We excluded studies not conducted in Brazil; prevalence-only studies without information on diagnostic pathways or care processes; genetic or neurobiological studies; intervention trials unrelated to diagnosis or care; and review articles, which were used only for contextual comparison. Articles published in English, Portuguese, or Spanish were considered eligible.

### 2.3. Information Sources and Search Strategy

A comprehensive literature search was conducted in the following electronic databases: PubMed/MEDLINE, Scopus, SciELO, and LILACS (via the Biblioteca Virtual em Saúde—BVS), as well as CAPES for grey literature. The search strategies used free-text terms across databases, supplemented by MeSH terms in PubMed, related to stigma, depression, anxiety, children and adolescents, and Brazil. The full search strategies for all databases are provided in Supplementary Annex I.

### 2.4. Study Selection

**Figure 1:**
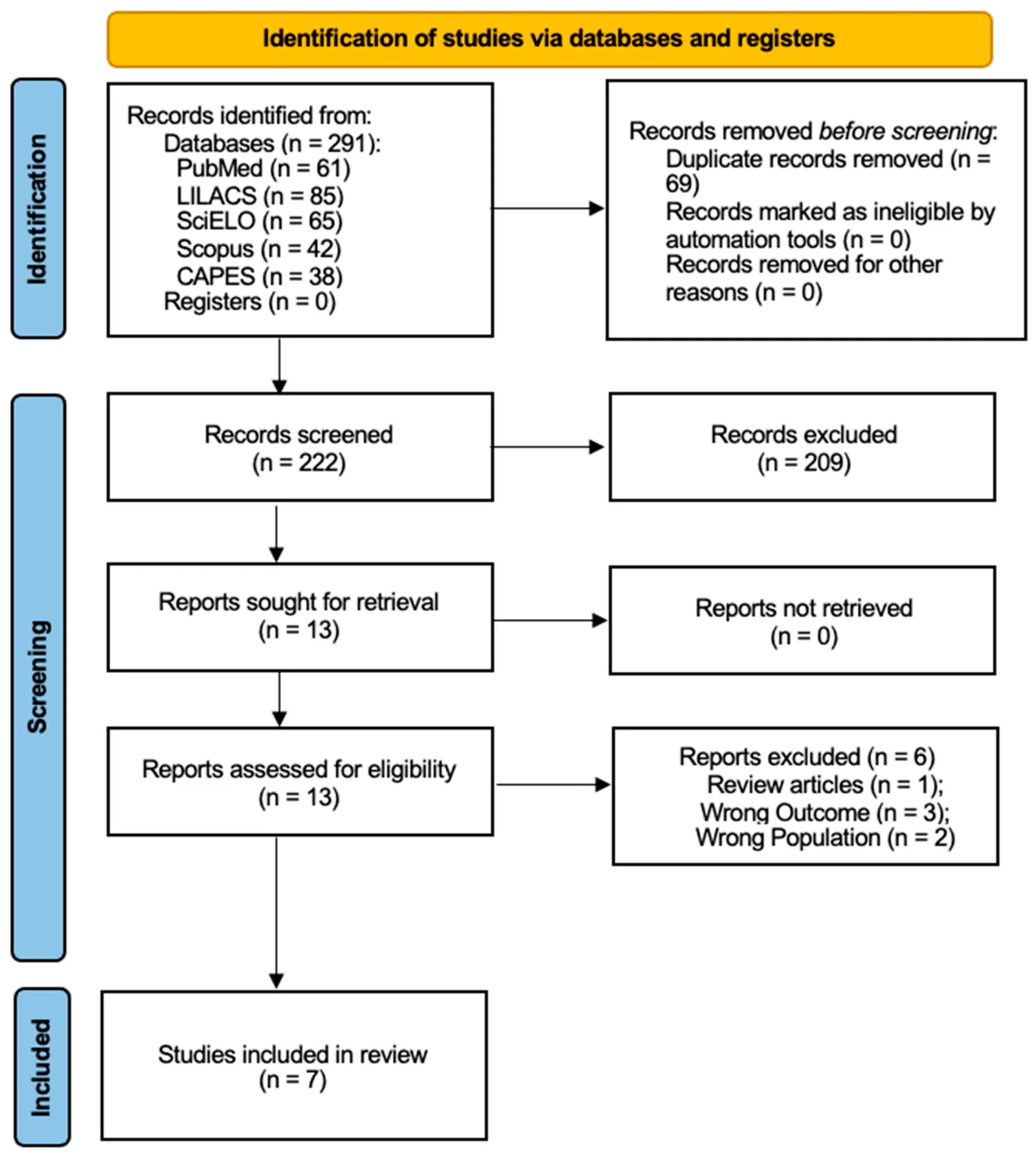
PRISMA-ScR flow diagram of the study selection process.

All identified records were exported to reference management software Zotero (version 9.0.6), and duplicates were removed prior to screening. Titles, abstracts, and full texts were independently screened by two reviewers (B.T.S. and M.E.O.H.). Discrepancies were resolved through discussion, and when consensus was not achieved, a third reviewer (M.G.C.F.) adjudicated. The study selection process is presented in the PRISMA-ScR flow diagram. The initial database searches were conducted on 12 April 2026; complementary searches of Scopus and CAPES were conducted on 25 August 2026 to broaden database coverage and identify the potentially relevant grey literature. These complementary searches were not specified in the registered protocol and are reported as a deviation; their records are included in the PRISMA-ScR flow diagram (Figure 1), and they yielded no additional eligible studies.

### 2.5. Data Extraction

Data were extracted using a standardized form. Extracted items included study characteristics (authors, year of publication, journal, study design, and sample size), geographic region, and demographic variables such as age, sex distribution, socioeconomic indicators (e.g., public vs. private healthcare use), parental education, and race/ethnicity, when available. Exposure variables included population and setting, types of stigma identified, primary stigma type, conceptualization or definition of stigma, and measurement tools used. Outcome variables included mental health outcomes (depression, anxiety, and suicidality), severity or clinical scores when reported, health system or coverage characteristics, and key findings regarding the association between stigma, clinical outcomes, and care-related processes.

Data were extracted by B.T.S. and verified by M.E.O.H. Stigma categories were assigned using a deductive approach based on predefined categories (public, structural, self-, and courtesy stigma), with study-specific findings examined for correspondence with each category. Structural stigma was distinguished from public stigma by its association with institutional practices, policies, or systemic barriers rather than interpersonal attitudes or discrimination. Differences in interpretation were resolved through discussion and consensus, with third-reviewer adjudication when necessary. Findings were synthesized narratively by stigma type, mental health outcomes, and care-related processes.

### 2.6. Methodological Appraisal

Methodological appraisal was conducted by B.T.S. using design-specific Joanna Briggs Institute (JBI) critical appraisal tools. Cross-sectional studies were assessed using the JBI Critical Appraisal Tool for Analytical Cross-sectional Studies [20], with judgments reported by domain as low, high, or unclear risk of bias. Qualitative studies were assessed using the JBI Critical Appraisal Checklist for Qualitative Research [21], with individual criteria rated as Yes, No, Unclear, or Not applicable. No numerical scores or percentage-based overall quality ratings were calculated. Detailed appraisal results are provided in Supplementary Annex II and Supplementary Tables S1, S1a and S1b.

An exploratory evidence strength appraisal was also conducted to support interpretation of the heterogeneous evidence across outcome domains. Evidence strength was considered narratively according to the evidence base, methodological limitations, consistency, directness, and evidence breadth. The appraisal framework and outcome-specific rationale are provided in Supplementary Annex III and Supplementary Table S2.

## 3. Results

### 3.1. Study Characteristics

The evidence base for this scoping review comprised seven studies with heterogeneous designs, including cross-sectional (*N* = 4) [3,16,22,23] and qualitative studies (*N* = 3) [10,24,25]. As detailed in Table 1, the included studies encompassed diverse populations, including school-based adolescents, transgender and non-binary youth, adolescent men who have sex with men (aMSM), and clinical samples of adolescents with major depressive disorder or suicide attempts.

Sample sizes ranged from small qualitative samples (*N* = 8) [10] to large cross-sectional samples (*N* = 1170) [16]. Geographically, the studies primarily focused on the South and Southeast, with additional coverage of the Northeast and one nationwide study. None specifically focused on the North or Midwest.

Among the included studies, quantitative evidence was primarily derived from larger cross-sectional samples [3,23], whereas the remaining studies provided qualitative or smaller-scale quantitative data, contributing mainly to contextual and care-related insights.

### 3.2. Types and Measurement of Stigma

Using the predefined deductive framework, stigma-related findings from the included studies were classified into four types: public, structural, self-stigma, and courtesy stigma (Table 2); these categories were not necessarily used by the original studies. Experiences of interpersonal discrimination or stigmatization were identified across the included studies, although the underlying reasons and assessment methods varied. Self-stigma was reported in five studies [3,10,22,24,25], particularly in relation to the internalization of mental health labels or gender-based stigma. Structural stigma was described in relation to institutional barriers, including racial discrimination affecting access to social and financial services and employment opportunities [23], and legal or systemic obstacles affecting transgender youth [22]. Qualitative evidence also identified courtesy stigma, referring to the social burden or stigma experienced by family members of affected adolescents [10,24].

Although all four stigma types were represented, their measurement and reporting varied across studies. Public stigma was the most frequently assigned category, while structural and self-stigma were more often inferred from reported experiences of discrimination or internalization processes. Importantly, the included studies examined both mental illness-related stigma and identity-based discrimination, which represent distinct but potentially overlapping phenomena.

The included studies can be further distinguished according to the focus of stigma or discrimination examined. Mental illness-related stigma was primarily addressed in studies 3 (Collins et al. [24]) and 7 (Viduani et al. [10]), particularly in relation to diagnostic labeling, disclosure, and help-seeking. Study 4 (Martins et al. [25]) addressed both mental health-related experiences and discrimination linked to marginalized identities. In contrast, studies 1 (Bittencourt et al. [16]), 2 (Chinazzo et al. [22]), 5 (Medeiros et al. [3]), and 6 (Santana et al. [23]) primarily examined interpersonal discrimination or stigma associated with social identities, including race, sexual orientation, and gender identity. This distinction concerns the focus of the exposure examined and is separate from the classification of stigma types presented in Table 2.

### 3.3. Stigma and Mental Health Outcomes

The included studies examined stigma and discrimination in relation to depression, anxiety, and suicidality (Table 3); quantitative associations were reported for depressive outcomes and suicidality, whereas anxiety was addressed only in qualitative studies. Perceived racial discrimination was associated with a higher prevalence of major depression (adjusted prevalence ratio [aPR] = 2.00; 95% CI: 1.37–2.97) [23]. Among adolescent men who have sex with men and adolescent transgender women, high-frequency (versus low-frequency) discrimination based on sexual orientation or gender identity, classified through latent class analysis of 15 indicators, was associated with severe depressive symptoms (adjusted Odds Ratio [aOR] = 1.71; 95% CI: 1.23–2.38) [3]. Separately, suicidal ideation was reported by 72.3% of transgender and non-binary participants in Chinazzo et al. [22]. This figure describes the sample and does not represent an association with discrimination; in that study, discrimination was not independently associated with suicidality after adjustment, whereas deprivation and depressive symptoms remained significant predictors [22].

Qualitative studies further described experiences linking peer-based discrimination to clinically significant distress, including reports of “intense distress” and hospitalization [25]. Overall, these findings were primarily derived from cross-sectional analyses, with effect estimates concentrated in a limited number of large-scale studies.

The magnitude of associations varied according to population characteristics and type of stigma exposure. Quantitative evidence was predominantly derived from studies examining discrimination-related stigma, particularly racial discrimination and discrimination based on sexual orientation or gender identity; differences in magnitude across exposures could not be formally compared. In contrast, qualitative studies provided insight into lived experiences of stigma, linking interpersonal discrimination to emotional distress, hospitalization, and suicidal behavior, although without quantifiable effect estimates.

### 3.4. Stigma and Mental Health Care Processes

The included qualitative studies described stigma-related barriers at multiple stages of the mental health care pathway (Table 3). Stigma was identified as a barrier to initial help-seeking and as a factor affecting diagnostic processes and disclosure [10,24,25]. Qualitative findings described a “secrecy” barrier, whereby adolescents may conceal symptoms to avoid being labeled “crazy” or “abnormal” [24].

Limited disclosure may also complicate recognition of acute mental health needs. In one qualitative study, 6 of 12 adolescents did not seek immediate hospital care following a suicide attempt; a direct link between this finding and stigma was not established [25].

Overall, evidence related to care pathways was limited to three qualitative studies, which described barriers involving help-seeking, diagnostic disclosure, and engagement with care across clinical and community settings. Across studies, stigma-related barriers were reported particularly in symptom recognition, disclosure, and engagement with care. Two of these studies [10,24] were conducted in Porto Alegre by overlapping research teams; because participant-level linkage was not reported, overlap between samples cannot be excluded, and their findings were not treated as independent confirmations.

### 3.5. Stigma Among Marginalized Populations

Stigma-related experiences were prominently reported among marginalized groups, including Black/Pardo youth and LGBTQ+ populations [3,22,23]. The included studies encompassed diverse socioeconomic and demographic profiles across educational, community, and healthcare settings (Table 1 and Table 3).

A limited number of studies have described the presence of multiple forms of stigma, including racial, regional, and gender-based discrimination [23,25]. These findings suggest that stigma exposures may overlap across social identities, although evidence on their combined effects on mental health outcomes remains limited.

### 3.6. Methodological Appraisal and Evidence Strength

The exploratory evidence strength appraisal indicated limited evidence strength for depressive outcomes, as well as for anxiety, help-seeking and care engagement, suicidality, and diagnostic processes and disclosure (Supplementary Table S2 and Supplementary Annex III). For depressive outcomes, only two studies reported adjusted effect estimates, both concerning identity-based discrimination rather than mental illness-related stigma. These judgments reflect methodological limitations, consistency, directness, and evidence breadth and do not represent formal GRADE certainty-of-evidence ratings.

Methodological appraisal identified selection concerns in three of four cross-sectional studies. Santana et al. [23] had the greatest number of high-risk domain judgments, involving selection and allocation, exposure classification, and outcome measurement. This warrants particular caution when interpreting its reported association between racial discrimination and major depression. Among qualitative studies, the main recurring limitation was insufficient researcher reflexivity in two studies (Supplementary Annex II and Tables S1, S1a and S1b).

### 3.7. Evidence Gaps

Several gaps in the literature were identified. The evidence base was limited by the predominance of cross-sectional and qualitative designs, which restrict causal inference. There was also an imbalance in study contributions, with a small number of large quantitative studies accounting for most effect estimates, while the remaining evidence was derived from smaller or qualitative samples.

A lack of standardized and culturally validated instruments to measure stigma among Brazilian youth was observed, with only one identified tool specifically addressing racial discrimination. In addition, geographic coverage was uneven, with no identified studies specifically focusing on the North or Midwest.

No included study evaluated interventions targeting stigma in relation to mental health care processes or examined its impact longitudinally across the care continuum, although intervention trials unrelated to diagnosis or care were outside the eligibility criteria of this review. Evidence on the integration of stigma into mental health care models and policies remains limited. Additional details of the evidence strength appraisal are provided in Supplementary Table S2 and Supplementary Annex III, with extended comparative analyses of previous reviews [14,15,26] presented in Supplementary Tables S3 and S4.

**Figure 2:**
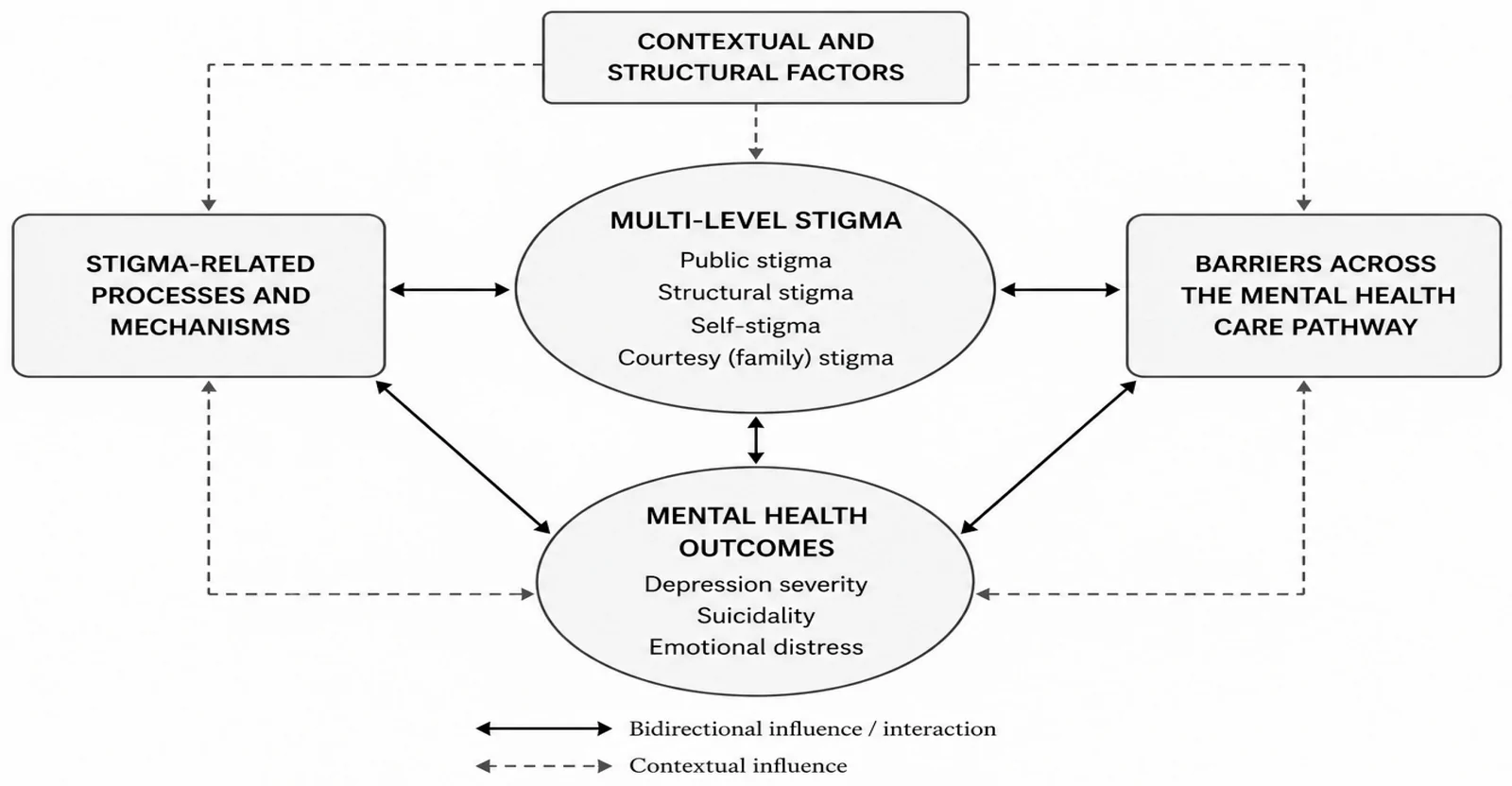
Authors’ proposed hypothetical conceptual model illustrating potential relationships between multi-level stigma, mental health outcomes, and barriers across the mental health care pathway among Brazilian youth.

Based on the findings of this review, we propose a hypothetical conceptual model illustrating potential relationships between multi-level stigma, mental health outcomes, and barriers across the adolescent mental health care pathway (Figure 2). The bidirectional arrows represent proposed interactions rather than empirically established causal relationships.

## 4. Discussion

This scoping review examined mental illness-related stigma and identity-based stigma and discrimination in relation to depression, anxiety, and mental health care processes among Brazilian youth, highlighting their relevance across multiple levels. While previous Brazilian studies have largely addressed stigma within broader discussions of social determinants of youth mental health, the present review brings together evidence on stigma as a multi-level construct associated with both clinical outcomes and care-related processes [15]. Our findings indicate that stigma and discrimination were associated with depressive outcomes, whereas associations with suicidality were not consistently retained after adjustment; qualitative studies also reported barriers to help-seeking, diagnostic processes and disclosure, and engagement with care. In particular, a small qualitative pilot study [10] highlights the potential role of “diagnostic stigma”, whereby receiving a mental health diagnosis may trigger self-stigma and “label avoidance,” potentially creating barriers to care engagement within the Brazilian health system [10,14].

These findings are broadly consistent with international and Latin American literature (Table S4) linking stigma to reduced help-seeking, poorer treatment adherence, and adverse psychosocial outcomes among children and adolescents [5,8,9,27]. However, while recent international reviews have increasingly incorporated structural dimensions of stigma and discrimination pathways [28,29], the Brazilian evidence synthesized in this review highlights reported barriers to care, including symptom concealment, delayed help-seeking, and difficulties accessing services. “Secrecy” emerged as a recurrent mechanism through which adolescents may avoid disclosure to protect themselves and their families from stigmatization and was described in contexts of delayed presentation and crisis-stage care, including suicide attempts [24,25]. These dynamics may present challenges for stigma-sensitive, community-based care within Brazil’s Psychosocial Care Network (RAPS), particularly in a health system marked by persistent regional inequalities [4,7,30].

A major challenge identified in this review relates to the limited availability of culturally validated stigma measures for Brazilian youth. Some studies relied on internationally developed instruments, which may not fully capture local distinctions between “estigma,” “preconceito,” and discrimination [3,16]. This measurement gap may limit the development of robust indicators for monitoring stigma and evaluating adolescent mental health policies within the Brazilian Unified Health System (SUS).

Our findings indicate that structural stigma and identity-based discrimination are relevant to mental health inequities among Brazilian adolescents marginalized by race, sexual orientation, or gender identity [3,22,23], although no included study examined their combined (intersectional) effects. The absence of studies from the North and Midwest also highlights geographic gaps in the evidence.

These findings are consistent with emerging international literature on structural stigma, although such inequities may be amplified in middle-income settings characterized by persistent social and regional disparities [5,28]. Together, the evidence suggests that addressing stigma in Brazil requires not only interpersonal interventions, but also equity-oriented policies and structural changes within mental health systems that may reduce barriers to care for marginalized youth.

### 4.1. Implications for Policy and Clinical Practice

Efforts to improve adolescent mental health outcomes in Brazil should address stigma across multiple levels, including structural barriers within the Psychosocial Care Network (RAPS), mental health literacy, and stigma-sensitive clinical practices.

These findings suggest that stigma-informed care may help address barriers to help-seeking, diagnosis, and treatment engagement, although the effectiveness of such approaches requires further investigation. This may include: (1) reducing stigma associated with receiving a mental health diagnosis in early care settings; (2) implementing inclusive and culturally sensitive care practices within specialized services, particularly for marginalized groups; and (3) strengthening school-based mental health literacy initiatives to address peer-related stigma.

These proposals should be regarded as hypotheses to be tested in intervention studies, including whether such approaches support earlier engagement with care and reduce diagnostic delays.

### 4.2. Limitations

This review has several limitations. The available evidence was predominantly cross-sectional and qualitative, limiting causal inference. In addition, the lack of standardized and culturally validated instruments for measuring stigma among Brazilian youth limits comparability across studies. A key challenge relates to the conceptual and linguistic overlap surrounding stigma in the Brazilian context, including the overlap between terms such as “preconceito,” “discriminação,” and “estigma” [3,16]. This measurement gap may contribute to underestimation of stigma-related outcomes and limit the development of robust indicators for policy evaluation. Furthermore, the majority of included studies comprised mixed adolescent and young-adult samples extending beyond the target age range, and age-stratified findings were not consistently available. Consequently, some extracted estimates reflect the full analytic samples and cannot be attributed exclusively to participants aged ≤17 years. The available studies also provided insufficient subgroup-specific evidence concerning Indigenous adolescents, rural populations, and adolescents with disabilities, limiting conclusions for these groups. Although children were eligible, no included study focused specifically on children, and the findings therefore primarily reflect adolescents and young adults. Two studies [16,23] were published before the establishment of the Psychosocial Care Network (RAPS) in 2011 [30]; their findings may not reflect the current organization of care, and interpretation of evidence collected across different periods warrants caution. In most quantitative studies, stigma or discrimination was examined as one of several exposures rather than as the primary construct. Methodological appraisal was conducted by a single reviewer. Finally, PsycINFO, Embase, and Web of Science were not searched, and reference lists were not hand-searched; additional terms (e.g., bullying or suicidality) might also have identified further studies.

### 4.3. Future Directions

Future studies should prioritize longitudinal designs, culturally adapted stigma measures, and interventions targeting stigma reduction across school, family, and healthcare settings. Additional studies from underrepresented Brazilian regions are needed.

## 5. Conclusions

This scoping review suggests that multi-level stigma is associated with adverse mental health outcomes, particularly depressive distress, while qualitative evidence identifies perceived barriers to help-seeking, diagnosis, disclosure, and care engagement among Brazilian youth. These associations occur within broader social inequalities involving race, socioeconomic disadvantage, sexual orientation, gender identity, and unequal access to healthcare. Integrating stigma-informed and equity-oriented approaches into the Psychosocial Care Network (RAPS) may support earlier engagement and recognition of mental health needs among marginalized youth, a hypothesis that requires evaluation. Despite the limited evidence base, these findings underscore the need for longitudinal research and culturally validated stigma measures to inform more equitable adolescent mental health policies in Brazil.

## Figures and Tables

**Table 1:** Detailed characteristics of included studies.

Code	Study	Study Design	Study Objectives	Population	Sample Size/Analytic *N*	Inclusion Criteria
1	Bittencourt et al. (2009) [16]	Cross-sectional	To estimate the prevalence and correlates of perceived discrimination among students.	7th-grade public-school students, Gravataí, RS	*N* = 1366; analytic *N* = 1170	All students present in 53 randomly selected 7th-grade municipal classrooms; ages 12–18 (79% aged 12–14; 21% aged 15–18); estimates reflect the full sample.
2	Chinazzo et al. (2023) [22]	Cross-sectional	To estimate suicidality and identify associated risk/protective factors among Brazilian transgender youth.	Transgender and non-binary youth recruited via online LGBTQ+ groups	*N* = 213; analytic *N* = 166	Self-identified transgender/non-binary; age 13–25 (mean 18.53 ± 2.50); age-stratified outcomes unavailable; estimates reflect the full mixed-age sample; informed consent provided
3	Collins et al. (2024) [24]	Qualitative	To examine barriers and facilitators to identifying adolescent depression across cultural settings.	Adolescents, parents, and professional stakeholders (Porto Alegre site)	Brazilian sample: *N* = 65 (11 adolescents; 54 adult stakeholders); 54 interviews and 2 focus groups.	Brazilian adolescents aged 14–17 (mean 15.4; *N* = 11); no age-related qualification needed. Findings also incorporate adult stakeholder perspectives.
4	Martins et al. (2026) [25]	Qualitative	To explore adolescents’ perceived reasons for suicide attempts through life narratives.	Adolescent survivors of suicide attempts at CAPSij, São Paulo	*N* = 67; analytic *N* = 12	Age 14–19 (41.7% aged 14–17); qualitative findings reflect the full mixed-age sample; suicide attempt recorded in SINAN/medical records (2020–2023)
5	Medeiros et al. (2023) [3]	Cross-sectional	To assess depressive symptoms and associations of violence/discrimination with severe depression among aMSM and aTGW.	aMSM and aTGW in three Brazilian capitals	*N* = 830; analytic *N* = 786	Age 15–19 (19.2% aged 15–17); age-stratified depressive symptom prevalence available, but adjusted association estimates reflect the full mixed-age sample; at least one sexual relationship with a man/trans person; nonreactive HIV test
6	Santana et al. (2007) [23]	Cross-sectional	To examine associations of perceived racial discrimination with major depression and depressive symptoms.	Children/adolescents in households, Salvador, Bahia	*N* = 1049; analytic *N* = 973	Age 10–21 (45.9% aged 10–17); age-stratified sample characteristics available, but association estimates reflect the full mixed-age sample; reported paid job or at least 8 h/week of domestic chores
7	Viduani et al. (2022) [10]	Qualitative	To explore adolescents’ experiences and understanding of a clinical diagnosis of depression.	Adolescents recently diagnosed with MDD at a university hospital	*N* = 10; analytic *N* = 8	Age 14–16 (mean age 15.3); recent diagnosis of depression; undergoing treatment at HCPA

Note: Abbreviations: aMSM = adolescent men who have sex with men; aTGW = adolescent transgender women; CAPSij = Centro de Atenção Psicossocial Infantojuvenil; FGD = focus group discussion; HCPA = Hospital de Clínicas de Porto Alegre; KII = key informant interview; MDD = major depressive disorder; RS = Rio Grande do Sul; SINAN = Sistema de Informação de Agravos de Notificação.

**Table 2:** Stigma mapping and categorization of included studies.

Code	Study	Population and Setting	Stigma Types Present (Standardized)	Primary Stigma Type	Stigma Definition/Conceptualization	Measurement Tool
1	Bittencourt et al. (2009) [16]	7th grade students (12–18 years), Southern Brazil	Public	Public	Broad definition encompassing bullying and family/community “mistreatment”	WHO Global School-based Student Health Survey
2	Chinazzo et al. (2023) [22]	Transgender/non-binary youth (13–25 years), Brazil-wide	Public, Structural (legal), Self-stigma	Public; Self-stigma	Minority Stress Theory: distal (discrimination) and proximal (internalized transphobia) stressors	Lifetime and Daily Discrimination Subscale
3	Collins et al. (2024) [24]	Adolescents/stakeholders, multi-country (Porto Alegre site)	Public, Structural, Self-stigma, Courtesy	Public	Stigma as a social barrier, including “label avoidance” and fear of being labelled “crazy”	Qualitative KIIs and FGDs
4	Martins et al. (2026) [25]	Suicide attempt survivors (14–19 years), São Paulo	Public, Structural, Self-stigma	Public	Intersecting violence/discrimination based on sexual orientation, gender, and regional origin	Oral life-history interviews; thematic analysis
5	Medeiros et al. (2023) [3]	aMSM and aTGW (15–19 years), 3 Brazilian capitals	Public, Structural, Self-stigma	Public	Minority Stress: daily stressors, perceived/internalized stigma, and violence	Latent class analysis based on 15 discrimination variables
6	Santana et al. (2007) [23]	Children and youth (10–21 years), Salvador	Public, Structural	Public	Racism as a “psychological pathogen” underlying mental health inequalities	4-item questionnaire on perceived racial prejudice and discrimination
7	Viduani et al. (2022) [10]	Adolescents with MDD (14–16 years), clinical setting	Public, Self-stigma, Courtesy	Public; Self-stigma	Diagnostic stigma: “social burden” and negative marking associated with a mental health label	Semi-structured interviews

Note: Abbreviations: aMSM = adolescent men who have sex with men; aTGW = adolescent transgender women; FGDs = focus group discussions; KIIs = key informant interviews; MDD = major depressive disorder; WHO = World Health Organization.

**Table 3:** Associations between stigma, mental health outcomes, and care pathways.

Code	Mental Health Outcomes	Assessment Instruments/Methods	Severity/Clinical Scores/Prevalence	Care Pathway	Health System	Key Findings	Demographic Context
1	Depression; Suicidality	Stigma: GSHS, single discrimination item. Mental health: GSHS-derived self-reported questions on sadness, loneliness, sleep difficulties, and suicidal ideation.	NR	NR	Public (Municipal)	Discrimination associated with greater suicidal ideation and depressive symptoms.	South; racially diverse sample; male/female; middle SES
2	Depression; Suicidality	Stigma: Intersectional Discrimination Index (InDI-D). Mental health: Modified Depression Scale (MDS); yes/no questions on suicidal ideation and attempts.	Depressive symptoms: 57.6%; suicidal ideation: 72.3%; suicide attempt: 42.7%	NR	NR	High suicidality; discrimination was not independently associated with suicidality after adjustment, while deprivation and depressive symptoms remained significant predictors.	Brazil-wide; race NR; transgender/non-binary; deprivation-based SES proxy
3	Depression; Anxiety; Suicidality	Stigma: Qualitative interviews and focus groups. Mental health: Clinical interviews; no standardized outcome scale.	Persistent (>2 weeks)	Help-seeking; access; diagnosis	SUS; Private	Stigma associated with “label avoidance,” concealment, and barriers to help-seeking and diagnosis.	South; race NR; male/female; mixed SES
4	Depression; Anxiety; Suicidality	Stigma: Oral life-history interviews. Mental health: Participant narratives; suicide attempts documented in SINAN and medical records. No standardized scale.	Intense psychological distress	Help-seeking; access; diagnosis	SUS (CAPSij)	6 of 12 participants did not seek immediate hospital care after a suicide attempt; stigma-related experiences emerged in narratives of care and abandonment.	Southeast; racially diverse sample; male/female/transgender; low SES
5	Depression	Stigma: Latent class analysis of 15 discrimination indicators. Mental health: Center for Epidemiologic Studies Depression Scale (CES-D), 20 items.	Severe depression: 37% aMSM; 45% aTGW	NR	NR	Discrimination associated with severe depression (aOR = 1.71; 95% CI 1.23–2.38).	Southeast/Northeast; racially diverse sample; aMSM/aTGW; mixed SES
6	Depression; Suicidality	Stigma: Four-item racial discrimination questionnaire. Mental health: Patient Health Questionnaire (PHQ), Portuguese version; separate questions on suicidality.	Major depression (DSM-IV)	NR	NR	Racial discrimination associated with major depression (aPR = 2.00; 95% CI 1.37–2.97) and suicidal ideation.	Northeast; racially diverse sample; male/female; mixed SES
7	Depression; Anxiety; Suicidality	Stigma: Semi-structured qualitative interviews. Mental health: K-SADS-PL (DSM-5); Children’s Depression Rating Scale–Revised (CDRS-R).	Mean CDRS-R: 49 (moderate–severe depression).	Help-seeking; access; diagnosis	SUS (university hospital)	Diagnostic stigma created a “social burden,” encouraging concealment and complicating engagement with care.	South; racially diverse sample; male/female; mixed SES

Note: Abbreviations: aMSM = adolescent men who have sex with men; aTGW = adolescent transgender women; aOR = adjusted odds ratio; CAPSij = Centro de Atenção Psicossocial Infantojuvenil; CDRS-R = Children’s Depression Rating Scale–Revised; CI = confidence interval; DSM-IV = Diagnostic and Statistical Manual of Mental Disorders, Fourth Edition; NR = not reported; PR = prevalence ratio; SES = socioeconomic status; SUS = Sistema Único de Saúde.

## Data Availability

No new data were created or analyzed in this study. Data sharing is not applicable to this article.

## Supplementary Materials

The following supporting information can be downloaded at https://www.mdpi.com/article/10.3390/healthcare14213616/s1, Supplementary File S1: Search Strategies; Supplementary File S2: Narrative Summary of Methodological Appraisal and Risk of Bias; Supplementary Fele S3: Exploratory Appraisal of Evidence Strength; Supplementary Table S1. Methodological Appraisal of Included Studies; Supplementary Table S1a: JBI Risk-of-Bias Assessment for Analytical Cross-sectional Studies; Supplementary Table S1b: Detailed JBI Critical Appraisal Checklist for Qualitative Research; Supplementary Table S2: Exploratory Evidence Strength Profile; Supplementary Table S3: Comparisons with Previous Reviews about Stigma in Brazil; Supplementary Table S4: Comparative Synthesis of Stigma Constructs and Mechanisms Across Previous Reviews.
