# Supplementary material for: Multi-Level Stigma, Discrimination, and Mental Health Care Barriers Among Brazilian Youth: A Scoping Review
Source: Healthcare (Basel). 2026 Oct 26;14(21):3616. doi: 10.3390/healthcare14213616 (PMC13763206; doi:10.3390/healthcare14213616)
Supplement: Supplementary file 1 [file healthcare-14-03616-s001.zip › healthcare-4603725-supplementary.pdf]

Supplementary File S1. Search Strategies

Initial database searches were conducted on 12 April 2026; complementary searches of Scopus and CAPES were conducted on 25 August 2026. Records retrieved: PubMed (n = 61), LILACS (n = 85), SciELO (n = 65), Scopus (n = 42), and CAPES (n = 38); total n = 291 (see PRISMA-ScR flow diagram).

1) PubMed

```
(
stigma[Title/Abstract] OR stigmatization[Title/Abstract] OR "social stigma"[Title/Abstract]
OR "self stigma"[Title/Abstract] OR discrimination[Title/Abstract] OR prejudice[Title/Abstract]
OR bias[Title/Abstract] OR stereotype[Title/Abstract]
OR "Mental Health Stigma"[Mesh]
)
AND
(
depression[Title/Abstract] OR depressive[Title/Abstract]
OR "depressive disorder"[Title/Abstract]
OR "depressive symptoms"[Title/Abstract]
OR anxiety[Title/Abstract] OR anxious[Title/Abstract]
OR "anxiety disorder"[Title/Abstract]
OR "anxiety symptoms"[Title/Abstract]
OR "Depressive Disorder"[Mesh]
OR "Anxiety Disorders"[Mesh]
)
AND
(
adolescent[Title/Abstract] OR adolescents[Title/Abstract] OR youth[Title/Abstract]
OR child[Title/Abstract] OR children[Title/Abstract]
OR "Adolescent"[Mesh] OR "Child"[Mesh]
)
AND
(
Brazil[Title/Abstract] OR Brasil[Title/Abstract] OR "Brazil"[Mesh]
)
```

2) LILACS (BVS)

```
(
stigma OR estigma OR estigmatização OR "estigma social" OR "autoestigma"
OR discriminação OR preconceito OR viés
)
AND
(
depressão OR depressivo OR "transtorno depressivo" OR "sintomas depressivos"
OR ansiedade OR ansioso OR "transtorno de ansiedade" OR "sintomas de ansiedade"
)
AND
(
adolescente OR adolescentes OR jovem OR jovens OR criança OR crianças
)
AND
```

(
Brasil OR Brazil
)

3) SciELO

Due to platform limitations, depression and anxiety searches were conducted separately in SciELO and combined during screening.

Depression

(stigma OR estigma OR estigmatização OR estigmatizacion
OR "social stigma" OR "autoestigma" OR "self stigma"
OR discriminação OR discriminacion OR discrimination
OR bias OR stereotype)
AND
(depression OR depressão OR depresion
OR "depressive disorder" OR "transtorno depressivo"
OR "depressive symptoms")
AND
(adolescent OR adolescents OR adolescente OR adolescentes
OR youth OR jovem OR jovens
OR child OR children OR criança OR crianças)
AND
(Brazil OR Brasil)

Anxiety

((stigma OR estigma OR estigmatização OR estigmatizacion
OR "social stigma" OR "autoestigma" OR "self stigma"
OR discriminação OR discriminacion OR discrimination
OR bias OR stereotype)) AND ((anxiety OR ansiedade OR ansiedad
OR "anxiety disorder" OR "transtorno de ansiedade"
OR "anxiety symptoms")) AND ((adolescent OR adolescents OR adolescente OR adolescentes
OR youth OR jovem OR jovens
OR child OR children OR criança OR crianças)
) AND ((Brazil OR Brasil))

4) Scopus

```
TITLE-ABS(
  stigma
  OR stigmatization
  OR "social stigma"
  OR "self stigma"
  OR discrimination
  OR prejudice
  OR bias
  OR stereotype)
AND
TITLE-ABS(
```

```
   depression
   OR depressive
   OR "depressive disorder"
   OR "depressive symptoms"
   OR anxiety
   OR anxious
   OR "anxiety disorder"
   OR "anxiety symptoms")
AND
TITLE-ABS(
   adolescent
   OR adolescents
   OR youth
   OR child
   OR children)
AND
TITLE-ABS(
   Brazil
   OR Brasil)
```

5) CAPES

The CAPES searches were conducted using the Catálogo de Teses e Dissertações da CAPES. Six simplified Boolean searches were performed without applying publication-year, language, or other search filters. No publication-year restriction was applied at any stage; language eligibility criteria were applied during screening. Simplified searches were used because the CAPES interface does not process complex Boolean strings; results were combined during screening.

```
estigma AND depressão AND adolescente
estigma AND ansiedade AND adolescente
preconceito AND depressão AND adolescente
preconceito AND ansiedade AND adolescente
discriminação AND depressão AND adolescente
discriminação AND ansiedade AND adolescente
```

Supplementary File S2. Narrative Summary of Methodological Appraisal and Risk of Bias

Methodological appraisal was conducted using design-specific Joanna Briggs Institute (JBI) critical appraisal tools. The four analytical cross-sectional studies were assessed using the revised JBI Critical Appraisal Tool for Analytical Cross-sectional Studies, and the three qualitative studies were assessed using the JBI Critical Appraisal Checklist for Qualitative Research. Methodological appraisal was used to inform interpretation of the evidence rather than study eligibility, and no study was excluded on the basis of appraisal findings.

For analytical cross-sectional studies, individual signalling questions were rated as Yes, No, Unclear, or Not applicable and synthesized into methodologically relevant domains: selection and allocation (Q1–Q2), exposure classification (Q3), outcome measurement (Q4), confounding (Q5–Q6), statistical conclusion validity (Q7), and reporting (Q8). Domain-level risk of bias was classified as low when all relevant signalling questions were rated "Yes," high when one or more were rated "No," and unclear when one or more were rated "Unclear" in the absence of a "No" judgment. No numerical score, percentage-based threshold, or overall methodological-quality classification was applied.

For qualitative studies, the 10 criteria of the JBI Critical Appraisal Checklist for Qualitative Research were retained as individual Yes, No, Unclear, or Not applicable judgments rather than converted into numerical scores or overall quality categories. The criteria assess congruity between the philosophical perspective, methodology, research question, data collection, analysis, and interpretation; researcher positioning and reflexivity; representation of participants and their voices; ethical conduct; and whether conclusions are supported by the analysis and interpretation.

Overall, the methodological appraisal indicated that the principal limitations of the cross-sectional evidence related to participant selection (study codes 2, 5, and 6) and, in one study, measurement of exposures and outcomes (study code 6). Santana et al. (study code 6) had high risk-of-bias judgments in three domains (selection and allocation, exposure classification, and outcome measurement), the largest number of high-risk domains among the assessed cross-sectional studies. Outcome measurement was also unclear in one study (study code 1). Confounding, statistical conclusion validity, and reporting were generally well addressed across the cross-sectional evidence. These limitations were considered particularly relevant when interpreting associations between stigma-related exposures and mental health outcomes,

given the cross-sectional nature of the evidence and consequent limitations in establishing temporality.

The qualitative evidence was generally methodologically coherent, with appropriate alignment between research questions, data collection, analysis, interpretation, and representation of participant perspectives. The principal recurring limitation was incomplete consideration of researcher influence and reflexivity (study codes 3 and 7), while the congruity between the stated philosophical perspective and methodology was unclear in these same studies. Study code 4 met all JBI qualitative appraisal criteria, although limitations associated with predominantly online data collection were considered when interpreting its findings.

A study-level summary of the principal methodological concerns is provided in Supplementary Table S1, with detailed domain-level judgments for cross-sectional studies in Supplementary Table S1a and item-level judgments for qualitative studies in Supplementary Table S1b.

Supplementary File S3. Exploratory Appraisal of Evidence Strength

Given the heterogeneity of the included evidence, a formal GRADE certainty-of-evidence assessment was not undertaken. Instead, an exploratory evidence strength appraisal was used to support interpretation of the mapped evidence across the principal outcome domains. This appraisal was descriptive and contextual and was not intended to provide formal certainty-of-effect ratings.

Evidence strength was considered across five dimensions: evidence base, methodological limitations, consistency, directness, and evidence breadth. The evidence base reflected the number and design of studies contributing to each outcome domain. Methodological limitations were informed by the design-specific JBI critical appraisal presented in Supplementary Tables S1–S1b. Consistency reflected the extent to which findings across contributing studies converged in direction or interpretation. Directness considered the extent to which study populations, stigma-related exposures or experiences, and outcomes addressed the review question. Evidence breadth considered the diversity and coverage of the contributing evidence, including study populations, settings, geographic representation, and the availability of complementary quantitative and qualitative evidence.

Overall evidence strength was derived through narrative consideration of these dimensions rather than numerical scoring or an algorithmic combination of domain judgments. Evidence was described as Moderate when findings were supported by multiple complementary studies, were generally consistent and substantially direct to the review question, and methodological or coverage limitations did not substantially undermine the overall pattern. Evidence was described as Limited when interpretation was constrained by methodological limitations, inconsistency, restricted evidence breadth, limited directness, or reliance predominantly on small or selected samples. These categories represent descriptive judgments concerning the relative strength of the evidence mapped in this review and should not be interpreted as formal GRADE certainty categories.

The evidence concerning depressive outcomes was considered Limited. Although findings across the contributing studies generally suggested associations between discrimination or stigmatizing experiences and depressive distress, only two studies reported adjusted effect estimates, both examining identity-based discrimination rather than mental illness-related stigma. The predominantly cross-sectional designs, methodological limitations, including multiple high-risk domains in Santana et al., heterogeneous populations and exposures, and limited direct evidence on mental illness-related stigma restricted the strength of the overall inference.

Evidence concerning help-seeking and care engagement was considered Limited. Although qualitative findings consistently described anticipated judgment, concealment, selective disclosure, and difficulties engaging others in obtaining care, the evidence was derived from small, selected urban or clinical samples, and no included quantitative study directly estimated the relationship between stigma and help-seeking or care engagement.

Evidence concerning suicidality was also considered Limited. Quantitative studies provided suggestive associations between discrimination and suicidal ideation, planning, or attempts, but these associations were not consistently retained after multivariable adjustment. Qualitative evidence provided complementary accounts of pathways involving discrimination, humiliation, social exclusion, hopelessness, and suicidal distress; however, heterogeneity in populations, stigma exposures, age ranges, and suicidality measures limited the strength of the overall inference.

Finally, evidence concerning diagnostic processes and disclosure was considered Limited. Qualitative studies consistently indicated that stigmatizing beliefs and anticipated negative reactions could affect recognition, acceptance, and disclosure of mental-health problems. However, the evidence was restricted to a small number of selected qualitative samples from specialized urban, clinical, or research contexts and did not permit estimation of the magnitude of stigma-related diagnostic delay.

Outcome-specific judgments and their supporting considerations are summarized in Supplementary Table S2. The methodological appraisal informing these interpretations is reported separately in Supplementary Annex II and Supplementary Tables S1–S1b.

Supplementary Table S1. Methodological Appraisal of Included Studies

Code	Study ID	Primary Study Design	Appraisal Tool	Key Concerns
1	Bittencourt et al., 2009	Cross-sectional (large sample)	JBI Cross-sectional	Mental health outcomes based on symptom indicators rather than diagnostic measures; cross-sectional design.
2	Chinazzo et al., 2023	Cross-sectional	JBI Cross-sectional	Convenience LGBTQ+ sample; potential selection bias relative to general population.
3	Collins et al., 2024	Qualitative	JBI Qualitative	Limited researcher reflexivity; recruitment restricted to selected study sites.
4	Martins et al., 2026	Qualitative	JBI Qualitative	Predominantly online interviews may have limited rapport, observation of non-verbal cues, and depth of disclosure.
5	Medeiros et al., 2023	Cross-sectional (large sample)	JBI Cross-sectional	Convenience recruitment, primarily through social media, with potential participation/selection bias.
6	Santana et al., 2007	Cross-sectional (large sample)	JBI Cross-sectional	Selection concerns; potential measurement bias in racial discrimination exposure and depression outcome assessment; cross-sectional design limits temporal inference.
7	Viduani et al., 2022	Qualitative	JBI Qualitative	Small clinical sample; interviews conducted shortly after diagnosis; limited researcher reflexivity.

Supplementary Table S1a. JBI Risk-of-Bias Assessment for Analytical Cross-sectional Studies

Code	Study ID	Selection & allocation (Q1–Q2)	Exposure classification (Q3)	Outcome measurement (Q4)	Confounding (Q5–Q6)	Statistical conclusion validity (Q7)	Reporting (Q8)
1	Bittencourt et al., 2009	Low	Low	Unclear	Low	Low	Low
2	Chinazzo et al., 2023	High	Low	Low	Low	Low	Low
5	Medeiros et al., 2023	High	Low	Low	Low	Low	Low
6	Santana et al., 2007	High	High	High	Low	Low	Low

Note: The revised JBI Critical Appraisal Tool for Analytical Cross-sectional Studies organizes signaling questions into domains relating to selection and allocation (Q1–Q2), classification of exposure (Q3), assessment/detection/measurement of outcomes (Q4), confounding (Q5–Q6), and statistical conclusion validity (Q7); Q8 assesses comprehensiveness of reporting. Domain-level risk of bias was classified as low when all relevant signaling questions were rated "Yes," high when one or more were rated "No," and unclear when one or more were rated "Unclear" in the absence of a "No" judgment. No numerical score or percentage-based overall quality rating was calculated.

Supplementary Table S1b. Detailed JBI Critical Appraisal Checklist for Qualitative Research

Code	Study ID	Q1	Q2	Q3	Q4	Q5	Q6	Q7	Q8	Q9	Q10
3	Collins et al., 2024	Unclear	Yes	Yes	Yes	Yes	Yes	No	Yes	Yes	Yes
4	Martins et al., 2026	Yes	Yes	Yes	Yes	Yes	Yes	Yes	Yes	Yes	Yes
7	Viduani et al., 2022	Unclear	Yes	Yes	Yes	Yes	Yes	No	Yes	Yes	Yes

Note: The JBI Critical Appraisal Checklist for Qualitative Research assessed 10 criteria rated as Yes, No, Unclear, or Not applicable. JBI items: Q1, congruity perspective/methodology; Q2, methodology/research question; Q3, methodology/data collection; Q4, methodology/data analysis; Q5, methodology/interpretation; Q6, researcher culturally/theoretically located; Q7, researcher influence addressed; Q8, participant voices adequately represented; Q9, ethical conduct/approval; Q10, conclusions supported by analysis/interpretation.

Supplementary Table S2. Exploratory Evidence Strength Profile

Outcome/domain	Evidence base	Methodological limitations	Consistency	Directness	Evidence breadth	Overall strength	Interpretation
**Depressive Outcomes**	4 cross-sectional + 1 qualitative (codes 1, 2, 4, 5, 6)	Temporality; selection and measurement limitations	Generally consistent	Mostly direct	Limited; heterogeneous exposures; two adjusted estimates	**Limited**	Findings suggest associations between identity-based discrimination and depressive outcomes, but adjusted estimates were limited to two studies; evidence on mental illness-related stigma remains sparse.
**Help-Seeking and Care Engagement**	3 qualitative (codes 3, 4, 7)	Small, selected samples; limited reflexivity in codes 3 and 7	Generally consistent	Direct to mostly direct	Limited; selected urban/clinical samples	**Limited**	Anticipated judgment, concealment and selective disclosure may impede help-seeking and care engagement.
**Suicidality (Ideation/Attempts)**	3 cross-sectional + 1 qualitative (codes 1, 2, 4, 6)	Temporality; selection and measurement limitations	Mixed but suggestive	Mostly direct	Moderate; heterogeneous populations and measures	**Limited**	Associations were not consistently retained after adjustment; qualitative evidence supports stigma-related pathways to suicidal distress.
**Diagnostic Processes and Disclosure**	3 qualitative (codes 3, 4, 7)	Small, selected samples; limited reflexivity in codes 3 and 7	Consistent	Direct	Limited; specialized urban/clinical contexts	**Limited**	Stigma influenced recognition, diagnosis acceptance and disclosure; evidence does not establish the magnitude of diagnostic delay.

Note: This exploratory evidence strength profile was developed to support interpretation of the heterogeneous evidence mapped in the review and does not constitute a formal GRADE certainty-of-evidence assessment. Evidence strength was considered narratively according to the size and diversity of the contributing evidence base, methodological limitations identified through the design-specific JBI appraisal, consistency across studies, directness to the review question, and breadth of populations and settings represented. Overall evidence strength was described as Moderate, or Limited, with greater methodological limitations, inconsistency, indirectness, or restricted evidence breadth resulting in a lower descriptive rating. No numerical scoring algorithm or formal certainty-of-effect classification was applied. The methodological appraisal informing these judgments is presented in Supplementary Tables S1–S1b.

Supplementary Table S3. Comparisons with Previous Reviews about Stigma in Brazil

Objective Domain	Sallum et al., (Present Review)	Marchionatti et al., 2024	Araújo et al., 2025	Melo et al., 2010	Conclusion/Gap
Stigma Mapping	Systematic mapping of four types: public, structural, self, and courtesy stigma.	Stigma noted as a risk factor but not systematically categorized.	Narrative focus on "social stigma" in general depression.	Focuses specifically on the "weight" of obesity-related stigma.	Sallum et al. provides a categorical mapping of four stigma types among Brazilian adolescents.
Stigma → Depression	Associations with depression presence and severity (aPR = 2.00; aOR = 1.71), derived from mixed adolescent and young-adult samples.	Confirms violence and racism increase depression risk.	Agrees that stigma intensifies symptoms and suffering.	Links weight-bias to depression and low self-esteem.	Consistent direction of findings; Sallum et al. reports study-level effect estimates.
Suicidality	Maps suicidality outcomes alongside stigma-related experiences; associations were not consistently retained after adjustment.	Reports high indigenous suicide rates but not as a stigma mechanism.	Mentions suicide as a possible consequence of social neglect.	Not a primary focus; mentions "tragic consequences".	Sallum et al. examines suicidality alongside stigma-related experiences.
Help-Seeking	Reported in qualitative studies as a barrier linked to "label avoidance" and secrecy.	Highlights gaps in service use but lacks stigma-specific analysis.	Strong emphasis: stigma prevents patients from seeking help.	Notes that bias keeps patients away from healthcare services.	Sallum et al. & Araújo align on help-seeking; Sallum adds "secrecy" nuance.
Diagnostic Processes	Explores how diagnostic labeling may contribute to self-stigma and secrecy.	Focuses on instrument psychometrics, not the stigma of labeling.	Superficial mention of diagnostic labeling as a barrier.	Notes that medical labels can trigger negative stereotypes.	Sallum et al. places particular emphasis on stigma associated with clinical diagnosis.
Access/Quality	Stigma may function as a barrier to care access and quality	Focuses on underfunding and systemic gaps in the RAPS/SUS.	Describes social stigma as a barrier to treatment entry.	Reports 48% of nurses feel uncomfortable treating obese patients.	Sallum et al. highlights how stigma-related barriers may intersect with access to care in the Brazilian context.
Engagement with Care	Stigma-related barriers were identified in help-seeking, disclosure, diagnostic processes, and engagement with care.	Not specifically addressed as a stigma-driven outcome.	Emphasizes that stigma reduces adherence and quality of life.	Notes that fear of exposure leads to refusal of medical exams.	Sallum et al. extends the comparison to disclosure, diagnostic processes, and broader engagement with care.
Intersectionality	Includes racial, sexual orientation, gender identity, and regional discrimination; combined (intersectional) effects were not examined.	Strong on Black/Indigenous violence but not "stigma categories".	Limited focus; primarily general adolescent population.	Explores cultural and family differences in obesity-bias.	Sallum et al. maps multiple stigma types; intersectional effects remain to be tested.
Methodological Coverage	Maps qualitative and quantitative evidence together.	Large-scale quant (734 studies); explicitly excluded qualitative.	Narrative review of 12 studies; lacks standardized synthesis.	Narrative review of 27 studies.	Sallum et al. combines qualitative and quantitative evidence within a single mapping.
Research Gaps	Identifies regional absence (North/Midwest) and care pathway "hidden numbers".	Notes lack of nationally representative clinical surveys.	Highlights need for more research across diverse demographics.	Notes lack of studies on obesity-stigma across different ages/sex.	Sallum et al. specifically flags the "Care Pathway" data gap in Brazil.

Note: Abbreviations: aOR = adjusted odds ratio; LGBTQ+ = lesbian, gay, bisexual, transgender, queer/questioning, and other sexual/gender minorities; aPR = prevalence ratio; RAPS = Rede de Atenção Psicossocial; SUS = Sistema Único de Saúde. Melo et al. (2010) is included as a cross-condition comparison illustrating how stigma associated with a physical health condition may affect psychological well-being and healthcare engagement; it is not presented as direct evidence of mental illness-related stigma.

Supplementary Table S4. Comparative Synthesis of Stigma Constructs and Mechanisms Across Previous Reviews

Review & Context	Types of Stigma Identified	Conceptualization of Stigma	Association with Depression/Anxiety	Key Mechanisms Identified	Contribution to Present Synthesis
Chukwuma et al., 2024	Public; Self; Associative	Stigma as labeling plus status loss and prejudice.	Associated with symptom severity and decreased functional status.	Reduced treatment adherence and significant delays in care.	Connects stigma to clinical adherence and care delays.
Ferrie et al., 2020	Public; Self; Layered (Intersectional)	Stigma as an "identity threat" affecting social belonging.	Social rejection is associated with adolescent depressive symptoms.	Damage to self-concept; fear of peer-group exclusion.	Validates the developmental impact of "identity threat" in youth.
Kågström et al., 2025	Public; Self; Behavioral; Structural	Clear distinction between attitudes (stigma) and actions (discrimination).	Consistently linked to adverse outcomes; notes causal complexity.	Significant barriers to help-seeking; pervasive treatment gaps.	Highlights "Structural Stigma" as a critical but under-researched gap.
Kaushik et al., 2016	Public; Self; Perceived	Stigma as social labeling and the desire for "Social Distance."	Self-stigma is associated with poorer psychological recovery.	Internalization of "dangerousness"; selective disclosure (secrecy).	Provides context for "secrecy" processes also described in the included Brazilian studies.
Mascayano et al. 2016 (Latin America/LMIC)	Public stigma, self-stigma, courtesy (family) stigma, and structural/institutional stigma.	A multi-stage social process involving labeling, stereotyping, and status loss, deeply rooted in regional power dynamics and health inequalities.	Stigma is described as an important barrier to recovery, creating a "vicious cycle" where social isolation exacerbates symptom severity and persistence.	Label avoidance (help-seeking delay), family burden (hiding conditions due to courtesy stigma), and social distancing in school and work environments.	Provides regional Latin American evidence suggesting that "secrecy" and "family conflicts" are consistent cultural patterns; highlights a research-to-service gap relevant to the Brazilian context
Elizalde-Resano et al., 2025	Public; Self; Structural; Associative	Multidimensional: cognitive, emotional, and behavioral components.	Stigma negatively correlates with overall health-related quality of life.	Low mental health literacy; peer-group stigmatization.	Provides a framework for the multidimensional nature of the present findings.

Note: Abbreviation: SUS = Sistema Único de Saúde.
